# Proposal to add a Note to Rule 8 of the International Code of Nomenclature of Prokaryotes to clarify the meaning of the term ‘stem’

**DOI:** 10.1099/ijsem.0.006527

**Published:** 2024-09-16

**Authors:** Aharon Oren, Marko Kostovski, Mark J. Pallen

**Affiliations:** 1 Institute of Life Sciences, The Hebrew University of Jerusalem, The Edmond J. Safra Campus, 9190401 Jerusalem, Israel; 2 Institute of Microbiology & Parasitology, Faculty of Medicine-Skopje, University Ss Cyril and Methodius in Skopje, 1000 Skopje, Republic of North Macedonia; 3 Quadram Institute Bioscience, Norwich Research Park, Norwich, UK; 4 University of East Anglia, Norwich Research Park, Norwich, UK

**Keywords:** compound names, International Code of Nomenclature of Prokaryotes, word stems

## Abstract

According to the Rules of the International Code of Nomenclature of Prokaryotes (ICNP) and its appendices, names of higher taxa are formed by the addition of the appropriate suffix to the stem of the name of the type genus, and word stems derived from Latin and/or Greek are combined to compound names by means of an appropriate connecting vowel. The way the word ‘stem’ is used in the ICNP differs from the meaning of this term in textbooks of Latin and Greek grammar. We therefore propose to add a Note to Rule 8, clarifying that the term ‘stem’ when used in the ICNP corresponds with that part of the word that does not vary among the forms of the noun in the oblique cases, i.e., cases other than the nominative, and which can be obtained by deleting the ending of the genitive singular.

The term ‘stem’ is used in two contexts in the International Code of Nomenclature of Prokaryotes (ICNP) [1,2], when clarifying the correct formation of scientific names:

- When discussing how names associated with ranks higher than genus are built from the name of a type genus by addition of a suffix.
- When describing the word components, typically derived from Latin or Classical Greek, that are used to build compound names.

In both instances, some complexity arises, as Latin and Greek are inflected languages, where the grammatical endings that are attached to a noun or adjective depend on its use in a sentence—a grammatical function indicated by the so-called ‘case’ of the word. In Latin and Greek, there are several cases, each serving a specific role within the sentence. Thus, the Latin word for ‘water’ is *aqua* when used as the subject of a sentence (nominative singular case), but *aquam* when used as the object (accusative singular case), or *aquae* when used to mean ‘of water’ (genitive singular case), and *aquarum* when used to mean ‘of waters’ (genitive plural case).

When building compound words, for anything other than the final word component, we use what the ICNP calls ‘the stem’, i.e. that part of the word that does not vary among the forms of the noun, which in this context is *aqu*-. Such stems are typically followed by a connecting vowel to aid pronunciation, so that from this stem, we can build genus names such as *Aquifex*, meaning ‘water maker’ or *Aquimonas*, meaning ‘a water monad’.

Added complexity arises because, for many Latin and Greek nouns and adjectives, the form of the word seen when used as the subject of a sentence—the so-called *nominative case*, which is the form listed as a head word in dictionaries—differs from that seen in all other contexts (in technical terms, in the *oblique cases*). Thus, if we wish to say ‘of the water maker’ or ‘of the water makers’ in Latin, we say *aquificis* or *aquificum,* which lack the ending *-ex* seen in the nominative form. Furthermore, we derive the stem used in word building by removing the grammatical endings from the forms of the word seen in these oblique cases, giving us in this case the stem *aquific-*. It is this form, rather than *Aquifex*, that is used to build the phylum name *Aquificota*.

The key point here is that the stem used in compound word formation is usually different from the head word seen in dictionaries or from the form used as a genus name, and it is often not possible to guess the stem from these forms. Instead, one must consult dictionaries, which list the forms of the word seen in the oblique cases, particularly the genitive singular. Table 1 shows examples for Latin and Greek nouns; similar grammatical rules apply to Latin and Greek adjectives. Once one knows these forms, then one can derive the stem by removing the grammatical endings.

**Table 1.** Examples of stems of Latin and Greek nouns and the way these are used to form compound names of prokaryotic genera

Dictionary listing	Genitive singular ending	Stem used in word formation without connecting vowel	Stem used in word formation with connecting vowel	Example
L. fem. n. *aqua, aquae,* water	*-ae*	*aqu-*	*aqui-*	*Aquifex*
L. masc. n. *limus, limi*, mud	*-i*	*lim-*	*limi-*	*Limibacillus*
L. masc. n. *fluvius, fluvii*, river	*-i*	*fluvi-*	*fluvii-*	*Fluviimonas*
L. neut. n. *solum, soli,* soil	*-i*	*sol-*	*soli-*	*Solibacter*
L. masc. n. *fons, fontis*, spring	*-is*	*font-*	*fonti-*	*Fontibacillus*
L. masc. n. *lacus, lacus,* lake	*-us*	*lac-*	*laci-*	*Lacibacterium*
L. fem. n. *glacies, glaciei,* ice	*-i*	*glacie-*	*glaciei-*	*Glacieibacterium*
Gr. fem. n. κρηνη, κρηνης (transliterated: *crene*, *crenes*), spring	*-es*	*cren-*	*creno-*	*Crenobacter*
Gr. masc. n. πηλος, πηλου (transliterated: *pelos*, *pelou*), mud	*-ou*	*pel-*	*pelo-*	*Pelobacter*
Gr. fem. n. ϑριξ, τριχος (transliterated: *thrix*, *trichos*), hair	*-os*	*trich-*	*tricho-*	*Trichodesmium*
Gr. neut. n. αἱμα, αἱματος (transliterated: *haema*, *haematos*), blood	*-os*	*haemat-*	*haemato-*	*Haematospirillum*

In most cases, differences between the stem and the head word involve only the ending of the word, e.g. *aquific-* versus *Aquifex or aquimonad-* versus *Aquimonas*. However, more extreme variation is possible, for example when using the latinized Greek word *thrix, trichos,* meaning hair, which gives rise to *Caldithrix* as a genus name, but to *Calditrichota* as a phylum name.

Some confusion arises because the way the term ‘stem’ is used in the ICNP and in guidelines for the formation of names of prokaryotes [3,7] differs from the way the term is used in traditional textbooks of Latin and Greek grammar [8,9]. In these settings, ‘stem’ is used to mean an idealized version of the word that often contains a vowel to guide formation of genitive plural. So, grammarians talk of e.g. *A-stems* or *I-stems* or *O-stems*. Thus, in this context, the stem for the Latin word for ‘water’ is the A-stem *aqua-*, associated with the genitive plural form *aquarum*, while the O-stem for *bacillus* would be *bacillo-*, giving us the genitive plural *bacillorum*, meaning ‘of bacilli’.

The way in which the ICNP uses stems for the purposes of word formation falls in line with current practice in the International Code of Zoological Nomenclature [10] where ‘the stem for the purposes of the Code is found by deleting the case ending of the appropriate genitive singular’ and in the International Code of Nomenclature for algae, fungi and plants [11], where ‘[a] noun or adjective in a non-final position appears as a compounding form generally obtained by *…* removing the case ending of the genitive singular’.

We propose to clarify the use of the term ‘stem’ in the ICNP and to avoid confusion with its use in Latin or Greek textbooks by adding the following Note to Rule 8:

***Note*** **. The term ‘stem’ when used in the ICNP corresponds with that part of the word that does not vary among the forms of the noun in the oblique cases, i.e., cases other than the nominative, and which can be obtained by deleting the ending of the genitive singular.**

## Abbreviations

ICNP: International Code of Nomenclature of Prokaryotes

## References

[1] Oren A, Arahal DR, Göker M, Moore ERB, Rossello-Mora R (2023). International Code of Nomenclature of Prokaryotes. Prokaryotic Code (2022 Revision). Int J Syst Evol Microbiol.

[2] Oren A (2023). Emendation of Rules 8, 15, 22, 25a, 30(3)(b), 30(4), 34a, and Appendix 7 of the International Code of Nomenclature of Prokaryotes. Int J Syst Evol Microbiol.

[3] Trüper HG (1999). How to name a prokaryote?: Etymological considerations, proposals and practical advice in prokaryote nomenclature. FEMS Microbiol Rev.

[4] MacAdoo TO, Goodfellow M, O’Donnell AG (1993). Handbook of New Bacterial Systematics.

[5] Oren A, Rainey FA, Oren A (2011). Taxonomy of Prokaryotes - Methods in Microbiology.

[6] Oren A (2019). Bergey’s Manual of Systematics of Archaea and Bacteria.

[7] Oren A, Li W-J, Jiao J-Y, Salam N, Narsing Rao MP (2024). Modern Taxonomy of Bacteria and Archaea: New Methods, Technology and Advances.

[8] Greenough JB, Kittredge GL, Howard AA, D’Ooge BL. (1903). Allen and Greenough’s New Latin Grammar for Scholars and Colleges.

[9] Goodwin WW (1900). A Greek Grammar.

[10] International Commission on Zoological Nomenclature (1999). International Code of Zoological Nomenclature, 4th ed. https://www.iczn.org/the-code/the-code-online/.

[11] Turland NJ, Wiersema JH, Barrie FR, Greuter W, Hawksworth DL (2018). International Code of Nomenclature for Algae, Fungi, and Plants (Shenzhen Code) Adopted by the Nineteenth International Botanical Congress, Shenzhen, China, July 2017.

